# Work‐Influenced Circadian Disruption Connected to Disease Risk but Not Microbiomes in a Cohort of Philadelphia Nurses

**DOI:** 10.1002/ajhb.70314

**Published:** 2026-08-06

**Authors:** Clare Super, Maira Asif, Charlene Compher, Theodore G. Schurr, Morgan K. Hoke

**Affiliations:** ^1^ Department of Anthropology University of Pennsylvania Philadelphia Pennsylvania USA; ^2^ Department of Biobehavioral Health Science University of Pennsylvania, School of Nursing Philadelphia Pennsylvania USA; ^3^ Department of Anthropology University of North Carolina North Carolina USA; ^4^ Carolina Population Center, University of North Carolina North Carolina USA

**Keywords:** cardiometabolic disease, circadian rhythms, precarity, schedules, work environment

## Abstract

**Objectives:**

Circadian rhythms influence activity cycles in humans, and circadian rhythm disruption (CRD) can negatively impact cardiometabolic disease risk and microbiome composition. This study documented CRD in a sample of nurses in Philadelphia, examining connections between CRD, disease risk, and work environment factors, proposing the concept of work‐influenced circadian disruption (WICD).

**Materials and Methods:**

A total of 75 nurses were recruited for the study. Disease risk indicators included triglycerides (TRG), C‐reactive protein (hs‐CRP), blood pressure (BP), and fecal gut microbiome composition. Surveys recorded CRD via sleep/exhaustion levels, weekly exercise, mealtime timing/length, shift diet, and night shift work. Work environment variables included staff/resources, physician‐nurse relations, break times, and patient care assignments. Mixed multiple regression models assessed CRD's associations with cardiometabolic disease risk indicators (H1) and work environment variables (H2). Microbiome similarity by CRD variables was tested using PERMANOVAs.

**Results:**

Lower exercise levels were associated with higher hs‐CRP (β = −4.2, *p* = 0.05), TRG (β = −9.8, *p* = 0.03), BP (β = −3.6, *p* = 0.01), and less break time (β = 0.1, *p* = 0.01). Higher exhaustion was linked to elevated hs‐CRP (β = 6.0, *p* = 0.02) and fewer staff/resources (β = −0.7, *p* = 0.01). Less mealtime was associated with higher BP (β = −0.2, *p* = 0.05) and shorter breaks (β = 0.2, *p* = 0.04). Night shifts were linked to higher BP (β = 6.3, *p* = 0.01). CRD variables were not significantly associated with microbiome profiles.

**Conclusions:**

CRD is associated with disease risk indicators and with work environment variables but not with microbiome profiles, suggesting these relationships may operate through other pathways. Further studies should explore biobehavioral networks in WICD.

## 1 Introduction

Circadian rhythm activity cycles are important for health (Sharma 2003) and their disruption can impact disease risk (Azami et al. 2019; Boivin and Boudreau 2014; Huang et al. 2022). Research within biological anthropology conceptualizes circadian rhythms as evolved adaptations shaped by natural environments (Pozzi et al. 2022; Rea et al. 2014; Samson et al. 2017) but often neglect how sociopolitical forces, such as exploitative work schedules and conditions, disrupt these biological systems in humans. This oversight is significant because, in circadian rhythm‐disrupting (CRD) work environments, people may experience significant irregularity in the eating, sleeping, and activity cycles that form their circadian rhythms. Focusing on nurses, a population burdened by shift work's demands, this study bridges the gap by examining CRD as a biocultural phenomenon: one where workplace power dynamics, staffing shortages, and constrained break times exacerbate CRD's cardiometabolic toll. Specifically, we focused on the way that work schedules may require workers to ignore their natural and evolved rhythms, leading to potential health consequences.

### 1.1 Circadian Rhythm Disruption and Health

Circadian rhythm refers to the natural oscillation of behavior and physiological functions that occurs roughly every 24 h (Young and Kay 2001). Organisms have both central and peripheral mechanisms that can respond to and be entrained by environmental cycles. These mechanisms coordinate biological processes to anticipate and prepare for regular change in order to better use resources (Sharma 2003).

Previous research has illustrated that humans' circadian rhythms are governed by light–dark cycles, social interactions, activity, stress, meal timing, and even temperature (Vitaterna et al. 2001). The sleep–wake cycle is the primary circadian rhythm. It is influenced mainly by light, which serves as a “Zeitgeber” or time‐giver, aligning body functions through the suprachiasmatic nucleus in the brain (Hastings et al. 2007). Consistent rhythms promote health, while disruption of the sleep–wake cycle has been linked to inflammation, hormone imbalances, and higher risks of metabolic and cardiovascular diseases (Reddy et al. 2024).

Food consumption also influences circadian cycles, with digestive and hormone‐related rhythms affected by regular feeding patterns. (Scheving 2000). Meal timing, nutritional quality, and alcohol/caffeine intake impact various systems, synchronizing peripheral rhythms and influencing metabolic outcomes (Abdollahi et al. 2021; Beaumont et al. 2004; Brager et al. 2010; Burke et al. 2015; Huang et al. 2010; Kim et al. 2021; Kohsaka et al. 2007). Even when controlling for sleep–wake cycles, disruptions in food‐related circadian rhythms are associated with metabolic diseases, cardiovascular issues, gastrointestinal conditions, and cancer (Charlot et al. 2021; Depner et al. 2018; Duboc et al. 2020; Palomar‐Cros et al. 2023; Srour et al. 2018).

The gut microbiome (GM) interacts with circadian rhythms, showing diurnal shifts in response to diet and light (Heddes et al. 2022; Lee et al. 2022; Thaiss et al. 2016; Zhang et al. 2023). The GM represents the collection of microbes that inhabit the human digestive tract, and its composition can be approximated via fecal sample (Hou et al. 2022). Host and GM rhythms interact bidirectionally. Dietary, sleep, and light input cause some species in the GM to signal the host epithelium by secreting metabolites known as microbial‐associated molecular patterns (MAMPs) (Vernocchi et al. 2016). MAMPs, in turn, affect host metabolism, immune function, and energy balance (Corbin et al. 2023).

Disruptions to the GM (dysbiosis) are associated with increased intestinal permeability (Di Vincenzo et al. 2024), gastrointestinal disorders (Bhattarai et al. 2017; Manor et al. 2020), inflammation (Al Bander et al. 2020), metabolic dysfunction (Hur and Lee 2015), cancer (Sepich‐Poore et al. 2021), and CVD (Witkowski et al. 2020). Robust examinations of circadian rhythms must thus consider this microbial influence, as it may explain contradictory findings and the variation in response exhibited by individuals under the same conditions.

### 1.2 Work Environment Influences CRD

Work environments influence CRD through several mechanisms. Among those factors affecting circadian stability include irregular sleep duration and timing, which is common among night shift workers, irregular schedule workers, and workers with unprotected time off (Buxton and Shea 2020; Chaput et al. 2020; Crain et al. 2020; Lecca et al. 2021; Litwiller et al. 2017). Job insecurity, high stress, long commutes, nightcaps, and irregular meals are also linked to disturbed sleep cycles (Crain et al. 2020; Salas‐Nicás et al. 2020; Shimura et al. 2020; Visvalingam et al. 2020). Night shift workers often experience irregular meal patterns, limited food availability, and missed meals, despite similar calorie intake to daytime workers (Bonham et al. 2016; Clark et al. 2023; Gifkins et al. 2018; Haus et al. 2016; Nyberg and Lennernäs Wiklund 2017; Strzemecka et al. 2014).

Long work hours correlate with poorer diet quality, skipped meals, and breakfast omission (Azami et al. 2019; Muff et al. 2011; Sam et al. 2019). Gig and precarious jobs are also associated with unstable eating habits, meal irregularity, and lower diet quality (Bohle et al. 2004; Dixon et al. 2014; Kong et al. 2019). These disruptions extend to the GM, with night shifts and stressful work events shown to contribute to GM dysbiosis (Mortaş et al. 2020; Gao et al. 2022).

### 1.3 Precarity Within Biocultural Anthropology and Political Economy

Informed by a critical biocultural approach (Goodman and Leatherman 1998; Hoke and Schell 2020), this project investigated how labor becomes embodied, i.e., how broader occupational‐environmental forces get under the skin of our crucial healthcare workers (Lock 2018). Here, we demonstrate that political‐economic conditions are not merely a backdrop but active drivers of circadian misalignment. By centering work as an embodied stressor, we argue that circadian health cannot be understood without detailing the structures that create work conditions.

We rely on the concepts of “precarious employment” or rising job insecurity and deteriorating working conditions as it has been applied as a social determinant of health to help us predict which work environment factors might affect workers' disease risk via CRD (Barbier 2011; Benach et al. 2014; Kreshpaj et al. 2020). Additionally, we use Hoke and Long's (2024) anthropological model of precarity to consider the temporal and intersectional nature of work environment stressors as they outline how uncertainty affects resource insecurity, allocation, and predictability. With these concepts in mind, we use the term “work‐influenced circadian disruption” (WICD) for the specific precarious work environments and work schedules that can contribute to CRD via sleep and meal schedule disruption/irregularity, reduction in time available for regular exercise activity, and decreased diet quality, leading to downstream health consequences.

Our overall goal is to identify WICD and its downstream disease risk effects in our study population of nurses. Further examination of WICD within this population will explore how precarity within the nursing workplace emerges from multilevel cycles of instability—from healthcare industry deregulation to worksite underinvestment. We ultimately conceptualize WICD not as an individual vulnerability but rather as a biosocial consequence of structural labor inequities.

Given the growing understanding of the relationship between work, stress, and dietary health, researchers have begun exploring how these variables affect healthcare workers who experience significant occupational demands while providing patient care. In particular, nurses have been subjects for researchers interested in precarious employment in the context of commitment to the job (Hult and Ring 2024), impact on self‐reported overall health (Hult et al. 2022), and the effects of the COVID‐19 pandemic on nurses' precarious employment status (Gunn et al. 2023; Llop‐Gironés et al. 2021). Thus, a cross‐sectional nurse sample was well suited to our study's WICD focus. Nurses work night and day shifts (Beebe et al. 2017), the job has documented influence on diet, exercise, and sleep habits (Beebe et al. 2017; Horton Dias and Dawson 2020), and some nursing positions have been associated with high stress levels and job insecurity (Happell et al. 2013; Hult et al. 2022).

**FIGURE 1:**
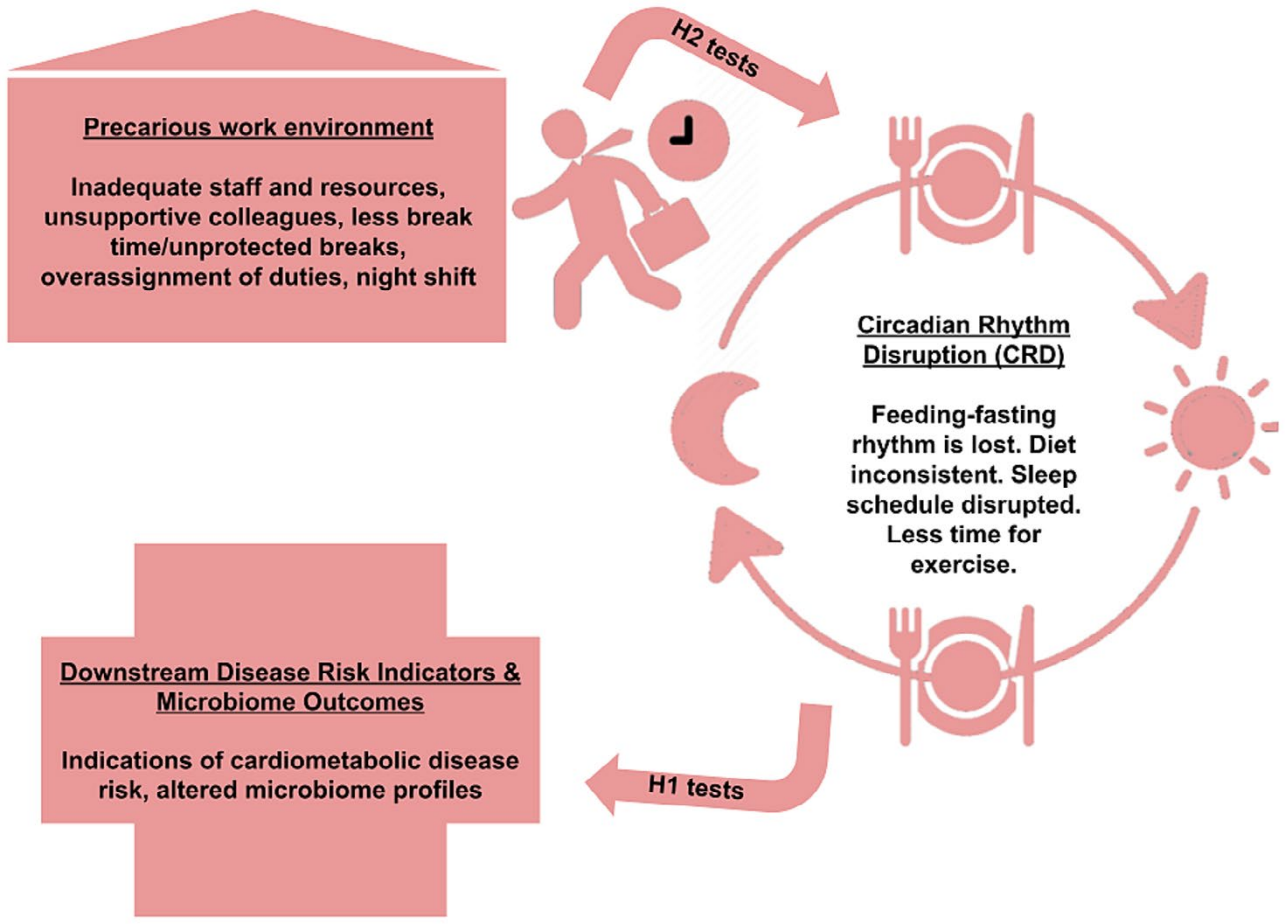
Schematic diagram of work‐influenced circadian disruption (WICD) and the analytical approach used to address our hypotheses. Our first hypothesis (H1) tests for associations between detected CRD and downstream disease risk indicators, while the second hypothesis (H2) tests for associations between precarious work environments and detected CRD.

This study used a prospective, observational approach and a cross‐sectional sample of nurses at a single hospital site. Our primary aim was to detect CRD and determine its associations with cardiometabolic disease risk indicators and microbiome variation. For Hypothesis One (H1), we theorized that CRD would occur within the sample and be associated with indicators of increased cardiometabolic disease risk and differential GM composition. Our secondary aim was to independently test if work environment precarity variables impacted the detected disruption. In Hypothesis Two (H2), we postulated that indicators of more precarious work environments would be associated with significant circadian disruption indicators. Figure 1 provides a schematic diagram for these hypotheses.

## 2 Materials and Methods

We collected biomarker and survey data from a cross‐sectional sample of nurses at Pennsylvania Hospital (PAH) via the Nurses' Occupational Health, Microbiome and Metabolism Study (NOHMMS) using a protocol approved by the University of Pennsylvania Institutional Review Board (IRB #850564). Participants were included if they were full‐time nurses, not pregnant, were trained in self‐collecting blood pressure measures, and had not recently taken antibiotics, antimetabolites, antipsychotics, or calcium‐channel blockers. Each participant signed an informed consent form during screening, which outlined confidentiality assurances and the voluntary nature of the study. The study sample consisted of 75 nurses from thirteen units and several positions, including staff, charge, advanced practice, coordinator, researcher, and case manager.

Participants completed a survey questionnaire, a 24‐h diet recall, and photographed all snacks and meals consumed over 24 h. Qualtrics online and mobile platforms (Seattle, WA) were used to securely collect survey data. Surveys were only accepted if they were filled out within the 24‐h collection period to ensure accuracy in describing shift conditions. Covariates investigated for potential impacts on participants' GMs and health included age, gender, ethnicity, body mass index (BMI), income, hospital role, nursing unit, and nursing license (Hult et al. 2022; Huttenhower et al. 2012; Miller et al. 2011; Niclou et al. 2018). Fecal samples and dried blood spots (DBS) were collected via take‐home “kits” (Crimmins et al. 2014; Qian et al. 2020). All materials were collected over a 24‐h period that included a shift at PAH. Participants were recruited via email listserv, posters, and word of mouth, and received $50 for biomarker kit completion and $25 for completion of surveys in pre‐paid debit cards.

We assessed cardiometabolic disease risk indicators using triglyceride (TRG), high sensitivity C‐reactive protein (hs‐CRP), and blood pressure (BP) measures. Low‐grade increased hs‐CRP levels indicated chronic stress/inflammation, higher hypertension risk and higher CVD risk (Backes et al. 2004; Romero et al. 2009). Increased TRG indicated higher risk of developing cardiometabolic disease (Miller et al. 2011). Increased systolic/diastolic BP levels indicated higher hypertension risk and higher CVD risk (*In Brief: What Is Blood Pressure and How Is It Measured?* 2019). GM profiles were created using 16S rRNA sequencing (Bokulich et al. 2018) (see details below). Work environment precarity was measured using subscales from the Practice Environment Scale of the Nursing Work Index (PES‐NWI) (Lake 2002) and details concerning work assignment and shift breaks.

### 2.1 Measures of Circadian Rhythm Disruption

CRD was evaluated with methods detailing nurses' feed‐fasting rhythms, diet quality, physical activity, sleep habits, shift types, and meal breaks. Exercise/activity was compared using a 4‐point descriptive rating scale scoring how many times a week participants participated in exercise or physical activity. The scale ranged from 1 (0–1 times per week) to 4 (6+ times per week). Sleep disruption/waketime exhaustion was assessed using a 5‐point scale scoring how often in the previous week participants had trouble staying awake during working hours due to disrupted sleep. The scale ranged from 1 (Never) to 5 (Every Day). The total time in minutes spent eating on meal breaks was reported directly by participants. Participants recorded the exact hours of their shifts and self‐identified status as “day” or “night” shift.

Participants photographed meals for a 24‐h period that included their shift and sent the photos in real time to the study coordinator, creating a time‐stamped log of meal timing and frequency. These data were recorded and minutes between meals averaged to create a metric for meal (or rather, eating incidence) frequency. Nutritional data were collected via 24‐h recall following participants' 24‐h collection period, referencing the photos of their meals, snacks, and drinks.

The study used the Automated Self‐Administered 24‐h (ASA24) Dietary Assessment Tool, a free, web‐based tool that enables automatically coded, self‐administered 24‐h diet recalls, also known as food diaries (Subar et al. 2012). Dietary Inflammation Index (DII) scores were calculated from ASA24 data. The DII score indexes the quantity of inflammatory components present in a diet, including individual foods, nutrients, and food components to estimate inflammatory potential in a diet. Negative scores indicate a less inflammatory diet; positive scores indicate a more inflammatory diet (Hébert et al. 2019).

### 2.2 Disease Risk Indicator Collection and Analysis

Participants' kits included extensive directions instructing proper self‐collection of DBS, fecal samples, and blood pressure measures. Participants were instructed to collect near the end or after a shift to ensure “exposure” to shift conditions. Systolic and diastolic BP were measured via aneroid sphygmomanometer. Participants were directed to take BP readings after sitting quietly for five minutes using PAH equipment at work. All participants were licensed nurses trained in blood pressure measurement as part of their clinical duties; standardized protocols were followed (*In Brief: What Is Blood Pressure and How Is It Measured?* 2019).

Participants self‐collected DBS, a method requiring a finger stick and collection of drops of blood on filter paper cards (Crimmins et al. 2014). DBS cards (Whatman protein saver) were dried and stored at −30°C before being cold shipped to the University of Washington Department of Laboratory Medicine and Pathology (UWLM) for processing and laboratory analysis. For the analyses, a 3.2 mm diameter disc was punched from each DBS for each biomarker measure into a 96‐well microtiter plate well using a BSD700 Semi‐Automated Dried Sample Puncher (BSD Robotics, Brisbane, Australia). Following elution, samples were analyzed following standard protocols for concentration of TGS and hs‐CRP in DBS eluates (for full protocol descriptions, see Supporting Information, 1.DBS Description of Assays). Hs‐CRP levels greater than 10 mg/L were excluded as they indicate an acute inflammatory event rather than chronic disease risk (Schakelaar et al. 2023).

DBS direct concentrations were converted into DBS plasma‐equivalent (PE) concentrations to evaluate the results based on clinically actionable decision points. To verify the plausibility of the DBS PE concentrations, the data were compared with results from analyses of conventional blood samples by NHANES in 2005–2020 (CDC and NCHS 2005). Each DBS PE concentration was within the respective NHANES range, indicating that sample integrity had not been obviously compromised during handling or shipping.

Fecal samples were self‐collected by participants (Globe Scientific 109 117 Stool Collection Tubes, Covidien Commode Specimen Collector 2400SA) and preserved in 95% ethanol, but not refrigerated, prior to delivery to the Biocultural Anthropology Methods Laboratory (BAMLab) for storage and processing. Participants were advised to collect samples near the end of their shift or after completing it, to capture potential “exposure” to shift conditions. Most samples were recovered within a few hours of collection. In the BAMLab, subsamples were frozen in a −80°C freezer.

Subsamples were cold shipped to the Amato Laboratory, in the Department of Anthropology at Northwestern University, where we used the laboratory's facilities to sequence the V4 region of the 16S rRNA gene to create GM sequence profiles. DNA was extracted (Qiagen PowerSoil PRO DNA Extraction Kit), and polymerase chain reaction (PCR) was used to amplify the V4 region of the 16S rRNA gene with 515f/926r primers (Walters et al. 2015). (For full protocol descriptions, see Supporting Information 2: GM DNA Extraction Protocol for Qiagen PowerSoil PRO DNA Extraction Kit).

Sequencing of barcoded amplicons was performed on an Illumina MiSeq (DNA Services Facility, University of Illinois‐Chicago) at a depth that allowed for at least 20 000 sequences per sample. Sequence data were processed using QIIME2 (Bolyen et al. 2019) and read pairs were processed to identify amplicon sequence variants with DADA2 (Callahan et al. 2016). Taxonomic assignments were generated by comparison to the Greengenes reference database (McDonald et al. 2012), using the naive Bayes classifier implemented in scikit‐bio (Bokulich et al. 2018). A phylogenetic tree was inferred from the sequence data using MAFFT (Katoh and Standley 2013). Similarity between samples was assessed by weighted and unweighted UniFrac distance (Lozupone et al. 2007).

### 2.3 Work Environment Measures

The staffing/resource adequacy and collegial nurse‐physician relations components of the nursing work environment were documented using key subscales from the PES‐NWI, which has been widely validated as a method for comparing nurses' work environments (Swiger et al. 2017). Adequacy in support services, time/opportunity to discuss patient care, and the number of nurses and staff were addressed in a four‐item index. Responses were scored on a 5‐point Likert scale ranging from 1 (strongly disagree) to 5 (strongly agree) and averaged. Higher average scores indicated more adequate resources and staffing.

Collegial nurse‐physician working relationships, joint practice between nurses and physicians, and the use of nursing diagnoses were assessed in a three‐item index. Responses were scored on a 5‐point Likert scale ranging from 1 (strongly disagree) to 5 (strongly agree) and averaged. Higher average scores indicated better nurse‐physician work relationships.

The rating of appropriateness of patient care assignment was addressed using a 5‐point Likert scale. Participants scored whether their patient assignment was appropriate, considering both the number of patients and the care they required, ranging from 1 (strongly disagree) to 5 (strongly agree), with higher scores indicating more appropriate patient assignments. The quality of meal breaks was tallied on a 3‐point descriptive rating scale ranging from 1 (I was not able to sit down for a meal break during my shift) to 3 (I was able to sit down for a meal break and was completely free of patient responsibilities). Higher scores indicated more protected breaks. The total length of all breaks was recorded directly in minutes.

### 2.4 Statistical Analysis

The statistical analysis of the models was carried out using R 4.1.5 software (R Core Team 2024). A two‐tailed alpha of 0.05 was used to determine statistical significance for all analyses. We estimated that a sample size of 69 was sufficient to detect a difference of 0.2 in UniFrac distance, assuming a standard deviation of 0.5 (PERMANOVA model, power = 80%, α = 0.05) using data from a similar study that investigated workplace and microbiome data (Karl et al. 2017). Based on a study that examined shift work and cardiometabolic disease risk indicators in night shift workers (Kwak et al. 2019), we determined sample sizes of 61, 50, and 73 were sufficient to detect a difference of 0.2 in TRG, hs‐CRP, and systolic BP measures, respectively, assuming a standard deviation of 0.5 (multiple regression model, power = 80%, α = 0.05). Calculations were run using the pwr and micropower packages (Champely et al. 2020; Vargas 2019). Cronbach's Alpha was used to assess internal consistency of the staffing subscale index (α = 0.858) and the nurse‐physician relations subscale index (α = 0.755).

To test H1 and determine associations between disease risk indicators and circadian rhythm disruption, three linear multiple regressions were run for hs‐CRP, TRG, and blood pressure, respectively, using the same circadian rhythm indicator variables: exercise per week, exhaustion level from lack of sleep, DII score, average time between meals, time spent on meal breaks, and day or night shift. Age was included as a covariate. Other covariates were included in linear regression models and were found to have no statistically significant associations; thus they were excluded for brevity. The variables of DII score and average time between meals had incomplete data that dropped the sample size below the power analysis sample size threshold. We dropped these variables and reran the models.

To test the GM associations of H1, permutational analysis of variance (PERMANOVA) tests were applied using the vegan package (Oksanen et al. 2024). This method uses weighted and unweighted UniFrac distances to identify differences in qualitative/richness community structure and quantitative/evenness community structure, respectively. between participants associated with CRD variables (Chen et al. 2012; Lozupone et al. 2007). Nonmetric multidimensional scaling (NMDS) visualized differences in beta diversity stratified by CRD variables.

To test H2 and determine if work environment precarity was connected to CRD, we identified some CRD variables that significantly associated with at least one disease risk indicator. These dependent CRD variables included exercise, sleep/exhaustion, and time spent on meal breaks. Shift time was included to account for when measures were taken. Covariates such as age or shift type were included in linear regression models and were found to have no statistically significant associations; thus they were excluded for brevity. We tested whether the independent work environment variables of staffing and resource adequacy, collegial nurse‐physician relations, time spent on breaks, patient care assignment appropriateness, and/or rating of shifts were associated with the CRD variables. Linear regression was used to assess the association of time on meal breaks, and ordinal logistic regression was used for the ordinal exercise and sleep/exhaustion variables.

## 3 Results

### 3.1 Demographic Covariates

A total of 75 PAH nurses (93.3% cis female) completed all survey and biomarker components of the study. Their mean age was 38 years (SD 12) and ranged from 22 to 67. The majority were white (88%, *n* = 66), staff nurses (75%, *n* = 56), earned between $100 000 and $200 000 annually (53%, *n* = 39), and held at highest, a registered nursing (RN) license (81%, *n* = 60) (Table 1). The demographic data indicated that the NOHMMS nurse participant cohort appeared skewed in several ways, which may impact interpretation of our models. When compared to a nationwide survey of nurses conducted biannually by the National Council of State Boards of Nursing (NCSBN) cis‐male and nonbinary nurses were underrepresented, salaries were high, and the median age skewed young (Smiley et al. 2023). By contrast, race was fairly representative of the national sample (Smiley et al. 2023).

**TABLE 1:** Demographic covariate summary statistics.

Variable	Category	*n*	Percent	Mean	Standard deviation
Age (Years)		75		37.5	12.1
Race	American Indian or Alaska Native	1	1.3		
	Asian	2	2.7		
	Black or African American	3	4		
	Other	2	2.7		
	White	66	88		
	Missing	1	1.3		
Gender	Cis Woman	70	93.3		
	Cis Man	5	6.7		
Income	$100000–$200 000	39	52		
	$50 000–$100 000	26	34.7		
	More than 200 000	9	12		
	Missing	1	1.3		
Hospital role	Advanced Practice Nurse	2	2.7		
	Charge Nurse	10	13.3		
	Coordinator of Clinical Program	2	2.7		
	Staff Nurse	56	74.7		
	Other Role	4	6.6		
Highest nursing license held	Advanced Practice License	6	8		
	RN License	60	80		
	Other	8	10.7		
	Missing	1	1.3		
Nursing unit	Behavioral Health	1	1.3		
	Dialysis	1	1.3		
	Emergency	4	5.3		
	Float women's health	5	6.7		
	Hematology/oncology	8	10.7		
	Intensive Care Nursery	8	10.7		
	Interventional Cardiology	1	1.3		
	Labor and Delivery	3	4		
	Medical Intensive Care Unit	11	14.7		
	Medical‐Surgical	14	18.7		
	Mother Baby Unit	9	12		
	Perianesthesia/post‐anesthesia care unit	6	8		
	Surgical Intensive Care Unit	4	5.3		

### 3.2 Descriptive Statistics

The greatest percentage of NOHMMS participants exercised 2–3 times per week (39%) and reported waketime exhaustion from disrupted sleep 1–2 times per week (49%). Overall, participants' diets trended slightly inflammatory with some outlying higher anti‐inflammatory scores (Mean = 0.117, Median = 0.3971, SD 2). The average NOHMMS nurse reported 205.2 min (3.42 h) between eating episodes (SD 14.16 min) and spent a total of 27 min (SD 13 min) eating meals at work. Nurses spent on average 20 min on breaks on their shifts (SD 17) and most nurses were able to sit but were not free of patient responsibilities on breaks (63%). Most participants were day shift workers (77%). For the PES‐NWI scores, the average Staffing and Resource Adequacy Score was 3.30 (SD 1.05) while the average Collegial Nurse‐Physician Relations Score was 3.60 (SD 0.97). For cardiometabolic disease risk indicators, the averages for hs‐CRP, TRG, and BP were within normal ranges (Table 2). Some participants were unable to complete BP measures so the n for BP is somewhat smaller (*n* = 71).

**TABLE 2:** Variable summary statistics.

Variable type	Variable	Category	*n*	%	Mean	Standard deviation
CRD variables	Exercise per Week	2–3	29	38.7		
		4–5	25	33.3		
		6+	2	2.7		
	Waketime Exhaustion (incidents per week)	Never	33	44.0		
		One to two times	35	46.7		
		Three to five times	3	4.0		
		Almost every day	3	4.0		
		Missing	1	1.3		
	Diet Quality (Diet Inflammatory Index)		69		0.1	2.0
	Average Minutes Between Eating		61		205.2	108.4
	Time Spent Eating Meals at Work (Min)		75		27.2	13.4
	Shift Type	Day	58	77.3		
		Night	17	22.7		
Work environment precarity variables	PES‐NWI Nurse‐Physician Relations		75		3.6	1.0
	PES‐NWI Staffing and Resource		75		3.0	1.1
	Patient Care Assignment Was Appropriate	Strongly disagree	2	2.7		
		Somewhat disagree	10	13.3		
		Neither agree nor disagree	8	10.7		
		Somewhat agree	22	29.3		
		Strongly agree	33	44.0		
	Break Quality Rating	Able to sit and free of patient responsibilities	12	16.0		
		Able to sit, not free of patient responsibilities	47	62.7		
		Unable to sit and take break	16	21.3		
	Break Time (Min)		75		20.3	16.6
Disease risk indicators*	hs‐CRP (mg/L)		75		7.4	15.9
	TRG (mg/dL)		75		101.9	30.0
	Systolic BP (mm Hg)		71		121.8	12.8
	Diastolic BP (mm Hg)		71		73.8	9.7

### 3.3 H1 Results: CRD Vs. Disease Risk Indicators and Microbiome

The results of the multiple linear regressions used to test the association of circadian disruption with disease risk variables are presented in Table 3 with additional findings in Supporting Information, (Table S1, Table 3). From the model excluding variables with missing data, more waketime exhaustion (β = 6, *p* = 0.02) and less exercise per week (β = −4.2, *p* = 0.054) were associated with higher hs‐CRP though less exercise was only marginally significant. Less exercise was associated with higher TRG (β = −9.8, *p* = 0.03). Less exercise (β = −3.6, *p* = 0.008), less time spent eating meals (β = −0.16, *p* = 0.052), and night shift work (β = 6.3, *p* = 0.013) were associated with higher BP, though less time spent eating was also only marginally significant.

**TABLE 3:** Health outcomes by circadian rhythm indicators (excluding variables with missing data).

Characteristic	hs‐CRP			Triglycerides			Blood pressure		
	Beta	95% CI^a^	*p*	Beta	95% CI^a^	*p*	Beta	95% CI^a^	*p*
Age (years)	−0.18	−0.49, 0.12	0.2	0.09	−0.52, 0.69	0.8	−0.16	−0.34, 0.02	0.079
Exercise incidents per week	−4.2	−8.5, 0.08	**0.054** *	−9.8	−18, −1.2	**0.026** **	−3.6	−6.2, −0.95	**0.008** ***
Waketime exhaustion (incidents per week)	6.0	1.1, 11	**0.016** **	2.7	−7.0, 12	0.6	−1.1	−4.0, 1.7	0.4
Time spent on meal breaks (min)	−0.04	−0.31, 0.23	0.8	0.00	−0.54, 0.54	> 0.9	−0.16	−0.32, 0.00	**0.052** *
Day or night shift									
Day	—	—		—	—		—	—	
Night	7.0	−1.4, 15	0.10	5.2	−12, 22	0.5	6.3	1.4, 11	**0.013** **
Sigma	14.9			29.9			8.73		
R squared	0.194			0.089			0.253		
No. obs.	74			74			71		

^a^ CI = Confidence Interval.

* *p* < 0.06.

** *p* < 0.05.

*** *p* < 0.01.

GM community composition was not significantly associated with any of the circadian disruption indicators or age for both weighted and unweighted UniFrac distances. NMDS demonstrated a lack of clustering by circadian disruption indicators (See Supporting Information, Table S2. Microbiome PERMANOVA Results Testing CRD Variable Associations with UniFrac Score).

### 3.4 H2 Results: Work Environment Precarity vs. CRD

Nurses who spent longer time on breaks reported more exercise occasions per week (β = 0.04, *p* = 0.01). Higher staffing and resource adequacy scores were associated with less waketime exhaustion (β = −0.74, *p* = 0.01). More break time was associated with longer time spent eating meals at work (β = 0.22, *p* = 0.03) (Table 4).

**TABLE 4:** Circadian rhythm outcomes by work environment predictors.

	Exercise (Ordinal Log. Reg.)^a^			Workplace exhaustion (Ordinal Log. Reg.)^b^			Time spent on meal breaks (Lin. Reg)^c^		
	Beta	*p*	95% CI	Beta	*p*	95% CI	Beta	*p*	95% CI
PES‐NWI staffing and resource adequacy score	−0.32	0.24	−0.86, 0.22	−0.74	**0.01** *	−1.33, −0.16	1.17	0.54	−2.56, 4.89
PES‐NWI collegial nurse‐physician relations score	−0.12	0.66	−0.66, 0.42	0.23	0.42	−0.32, 0.77	0.35	0.85	−3.32, 4.02
Time spent on breaks on shifts	0.04	**0.01** *	0.01, 0.07	−0.02	0.26	−0.05, 0.01	0.22	**0.03** *	0.02, 0.43
Patient care assignment appropriateness rating	0.15	0.50	−0.28, 0.57	0.25	0.27	−0.2, 0.7	0.42	0.78	−2.55, 3.39
Rating of shifts	0.45	0.27	−0.35, 1.26	0.64	0.16	−0.25, 1.53	−0.64	0.82	−6.26, 4.98

^a^ Residual Deviance: 164.01, AIC: 180.01.

^b^ Residual Deviance: 135.1565, AIC: 151.1565.

^c^ Sigma: 13.24, R2: 0.09.

* *p* < 0.05.

## 4 Discussion

**FIGURE 2:**
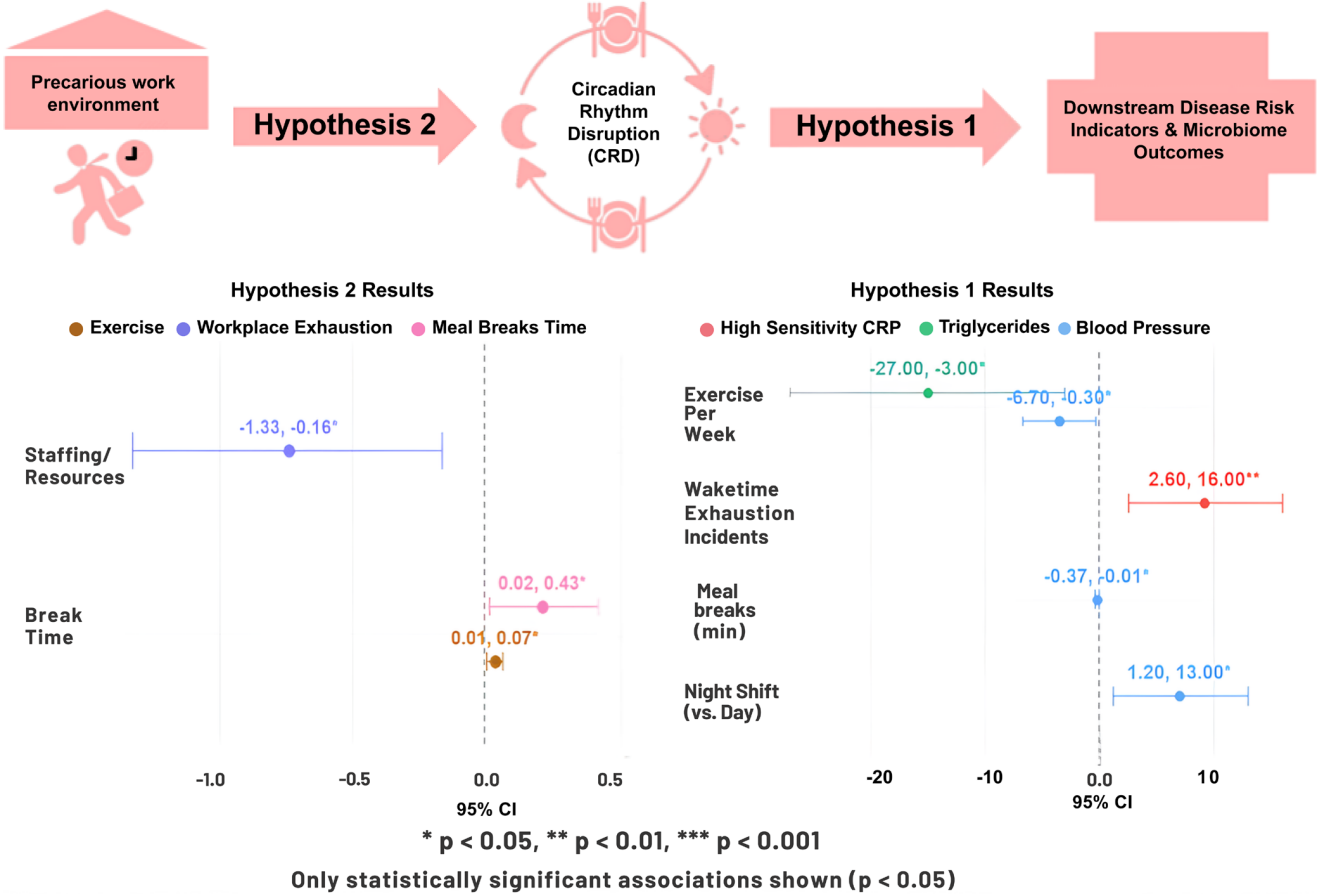
Hypothesized pathways and corresponding significant regression coefficient estimates. The upper panel illustrates the proposed conceptual model, in which precarious work environments are posited to influence circadian rhythm disruption (CRD), demonstrating work influenced circadian disruption (WICD) (H2). WICD was in turn hypothesized to drive downstream cardiometabolic disease risk and differential gut microbiome (GM) composition (H1). The lower panel presents coefficient plots for each pathway, displaying point estimates and 95% confidence intervals from separate regression models. Supporting H2, staffing and resource inadequacy were positively associated with workplace exhaustion, whereas break duration was associated with increased exercise engagement and time spent on breaks. Supporting H1, weekly exercise frequency was inversely associated with triglycerides and blood pressure, wake‐time exhaustion was positively associated with high‐sensitivity C‐reactive protein (hs‐CRP), and both meal breaks and night shift status were associated with blood pressure.

Our models show that participants who reported less break time also experienced less exercise (H2), which in turn increased their cardiometabolic indicators of disease risk (H1). Reporting fewer staff/resources was associated with higher exhaustion scores (H2), which was also associated with a cardiometabolic indicator of disease risk (H1). Shorter breaks corresponded to shorter mealtimes (H2), which additionally corresponded with a cardiometabolic indicator of disease risk (H1) though this relationship was only marginally significant (*p* = 0.052). Additionally, night shift work was associated with increased blood pressure. By contrast, CRD variables did not show a relationship with microbiome profiles (H1). Taken together, this study presents an initial assessment showing that nurses' cardiometabolic health may be susceptible to WICD. Independently, we demonstrated that nurses' work environment is associated with some markers of CRD. See Figure 2 for visualization that integrates the conceptual hypotheses outlined in Figure 1. with coefficient plots showing significant results from our models (marginally significant results excluded).

As less exercise per week was associated with higher hs‐CRP, TRG, and BP, it is likely that physical activity may be one entraining force that leads to increased disease risk upon disruption. However, it is important to note that the relationship between exercise and hs‐CRP was only marginally significant. Other studies have identified exercise as a powerful Zietgeber for training skeletal muscle clocks, influencing molecular clocks, negating some of the deleterious effects of disrupted sleep, and generally protecting metabolic health (for review, see Gabriel and Zierath 2019).

Longer break time was associated with more exercise per week. It is possible that participants were engaged in physical activity *during* their breaks or, instead, simply had more energy to exercise outside work if their shift contained more breaks. In either case, previous studies have demonstrated an increase in physical activity and downstream health benefits with more work breaks (de Bloom et al. 2017; Michishita et al. 2017; Nejati et al. 2016).

Higher hs‐CRP was associated with increased waketime exhaustion, suggesting a potentially important relationship between sleep, physical activity, and systemic inflammation. These findings align with previous studies that determined circadian misalignment is associated with increased inflammation (Meier‐Ewert et al. 2004; Morris et al. 2015; Weinert and Gubin 2022). Increased waketime exhaustion was also associated with lower staffing and resource adequacy scores. Studies have connected staffing and resource inadequacy to both disrupted sleep and waketime exhaustion in nurses and identified the sources of sleep disruption as increased stress, over‐assignment of duties, and longer working hours (Farag et al. 2021; Hong and Lee 2020; Stimpfel et al. 2022).

Our participants' BPs were associated with less exercise, less time spent eating meals (though only marginally significant), and night shift work. The increased BP may have multiple causes (Morris et al. 2016). Blood pressure immediately responds to stress but is also a fairly accurate indicator of cardio‐metabolic disease risk (Muntner et al. 2019). Shorter meal breaks, disrupted physical activity schedules, and night shift work have been extensively documented to be associated with increased BP (Lowden et al. 2010; Shen et al. 2023; Toffoli et al. 2023).

Finally, no circadian disruption variables influenced GM composition within our sample. The microbiome's role in circadian rhythm entrainment and downstream cardiometabolic disease risk is likely complex (Frazier and Chang 2020) and further research is needed to understand its role in WICD. The comparison of beta‐diversity between participants by circadian disruption variable used in this study represents one metric for investigating participants' GMs (Kers and Saccenti 2022). We intend to further investigate this relationship using other more complex metrics in future publications. Additional research focusing on the GM and WICD may require a more longitudinal approach as GM research benefits greatly from within‐participant comparison (Bodein et al. 2019).

This study has some limitations that are worth noting. First, the nature of this general cross‐sectional study, the multifactorial nature of the accompanying models, and the complexity of our variable interactions were not conducive to a mediation analysis necessary to further explore the relationships occurring within WICD. However, our study does provide a foundation for future research.

Second, this study relied primarily on indirect, qualitative, and self‐reported indicators of circadian function (e.g., self‐reported sleep duration, exercise frequency). Although actigraphy was incorporated, the resulting data lacked sufficient robustness for inclusion in circadian models. Future investigations should employ more precise biometric measures, such as functional actigraphy aligned with American Academy of Sleep Medicine guidelines (Smith et al. 2018), as well as complementary metrics like thermometry (Ortiz‐Tudela et al. 2010) and circadian ambulatory monitoring (Martinez‐Nicolas et al. 2019).

Additionally, the sample size may have been underpowered to detect associations that a more powered study could identify. Two of our CRD variables—DII and time between meals—suffered from missing data and were dropped from the more robust model. We also did not recruit a sufficient number of night shift workers to meet our power analysis threshold and compare to day shift workers. In addition, GM research is more able to detect differences in longitudinal studies when using shotgun analysis, as compared to 16S rRNA sequencing (Allaband et al. 2018).

Follow‐up studies with more participants, the analysis of fewer variables, and longitudinal experimental design could potentially reduce model errors and better determine the explanatory nature of the variables. Caution should be taken in generalizing these study results before they are validated using similar approaches and working populations.

This study drew together a burgeoning focus of research within human biology research—circadian rhythm health (Niclou et al. 2018; Samson 2021)—with a unique perspective on work/economic health impacts, a lens that has defined and redefined human biological research (Goodman and Leatherman 1998; Levin et al. 2011). It is significant that we were able to identify associations indicating WICD within this sample. This result suggests that future researchers may consider applying the WICD model to a variety of populations that could be experiencing disrupted schedules due to a precarious work environment.

Researchers need not search far for these populations. According to the American Psychological Associations' Work in American Survey, 57% of Americans are experiencing negative impacts from work‐related stress and work conditions (APA 2021). Researchers may even wish to use similar methods to examine populations facing other forms of precarity, as outlined in Hoke and Long (2024).

Moreover, human biology researchers may seek to investigate upstream political‐economic causes of WICD. This study relied on concepts of precarious employment (Benach et al. 2014). Through our research, we are developing a model of the Cycle of Precarious Employment (COPE), which offers a critical framework for future research by mapping how WICD emerges from cyclical precarity across multiple levels—from industry deregulation to worksite underinvestment—before manifesting in individual health disparities (Hoke and Long 2024). By applying this lens, human biologists can move beyond documenting WICD as an isolated phenomenon and instead trace its roots to structural labor inequities. Ultimately, COPE reframes WICD not as a ’lifestyle’ consequence but as a biosocial outcome of political‐economic systems, urging interventions that target circadian health at institutional, rather than individual, scales.

Finally, this study joins a plethora of research on the nursing work environment as interest in improving conditions for nurses to slow burnout and attrition rates has increased (Aiken et al. 2011; Barker and Nussbaum 2011; Skela‐Savič et al. 2023). Our findings could help administrators and nursing leaders pinpoint interventions to preserve circadian rhythms and downstream disease risk and suggest that particular attention should be applied to allowing time for physical activity routines.

## Author Contributions

**Clare Super:** conceptualization (lead), obtained funding, collected samples and data, processed samples, analyzed data, and wrote the paper. **Maira Asif:** collected samples and data, processed samples, analyzed data. **Charlene Compher:** conceptualization (support), reviewed/edited the manuscript, and contributed to the discussion. **Theodore G. Schurr:** conceptualization (support), reviewed/edited the manuscript and contributed to the discussion. **Morgan K. Hoke:** conceptualization (lead), obtained funding, reviewed/edited the manuscript, and contributed to the discussion, assisted with revisions.

## Funding

This work was supported by the National Science Foundation [BCS‐2147647], the Wenner Gren Foundation (DFG 584233), the BAM Lab, and Penn Anthropology research funds.

## Ethics Statement

This study was conducted in accordance with the principles of the Declaration of Helsinki. The research protocol for the Nurses' Occupational Health, Microbiome and Metabolism Study (NOHMMS) was reviewed and approved by the University of Pennsylvania Institutional Review Board (IRB #850564). All participants provided written informed consent during screening, which outlined confidentiality assurances and the voluntary nature of the study.

## Conflicts of Interest

The authors declare no conflicts of interest.

## Supporting information


**Table S1:** Health Risk Indicators by Circadian Rhythm Indicators (All Variables Included).
**Table S2:** Microbiome PERMANOVA Results Testing CRD Variable Associations with UniFrac Score.

## Data Availability

The data that support the findings of this study are openly available in Dryad at https://doi.org/10.5061/dryad.6q573n6dw.
