# Supplementary material for: Work‐Influenced Circadian Disruption Connected to Disease Risk but Not Microbiomes in a Cohort of Philadelphia Nurses
Source: Am J Hum Biol. 2026 Aug 6;38(8):e70314. doi: 10.1002/ajhb.70314 (PMC13444217; doi:10.1002/ajhb.70314)
Supplement: Supplementary file 1 [file AJHB-38-e70314-s001.docx]

# SUPPORTING INFORMATION

## 1. DBS Description of Assays

Description of assays performed at the University of Washington Department of Laboratory Medicine and Pathology (Seattle, WA; UWLMP) on dried blood spot (DBS) samples collected by the University of Pennsylvania Nurses Occupational Health, Microbiome, and Metabolism (NOHMMS) Study

**Methodology**

Gold-standard assays were performed on liquid (i.e., blood; serum and/or plasma) specimens to establish the concentrations of the analytes of interest to the Study in the assay calibrators, quality control (QC) samples, and assay validation samples used in the DBS assays. Total cholesterol (CHO), triglycerides (TRG), HDL cholesterol (HDL), and C-reactive protein (CRP) were measured on an AU680 Chemistry Analyzer (Beckman Coulter, Miami, FL). Hemoglobin A1c (HbA1c) was measured by immunoturbidometry on a DxC 600 (Beckman Coulter, Brea, CA

The dried blood spot (DBS) assays for CHO, TRG, and HDL were modifications of the gold-standard methods. In brief, an aliquot of DBS eluate was incubated with an assay-specific enzyme to produce hydrogen peroxide, which then reacted with a fluorophore and horseradish peroxidase (HRP) to produce fluorescence, measured by a spectrophotometer (BioTek Synergy HT, Agilent, Santa Clara, CA), in proportion to the concentration of analyte in the sample. The DBS assay for CRP was a modified version of a standard ELISA. Briefly, an aliquot of DBS eluate was incubated in a reaction plate coated with a CRP-specific antibody. After incubation, excess antibody was removed by washing, detector antibody coupled with HRP was added, excess HRP was washed away, and a chromogenic substrate was added. The light emitted in proportion to the CRP concentration in the sample was measured by the spectrophotometer. HbA1c was measured in DBS eluate using the DxC 600 standard protocol.

**Assay Details**

The assays on Study DBS were performed using validated protocols. CHO, HDL, and TRG were measured in duplicate by performing separate assays of two 3.2mm diameter dried blood-saturated filter paper discs punched from a DBS. HbA1c and CRP were measured in separate single 3.2mm discs. It was not possible to run all assays on each Study DBS due to some cards having limited specimen.

The concentrations of each analyte of interest were measured in matched DBS and liquid assay validation specimens. Liquid concentrations were regressed on DBS concentrations by ordinary least squares to establish a liquid-equivalent conversion equation for each analyte. The conversion equations were used to convert the analyte values in the Study DBS into liquid-equivalent values, which may then be evaluated using metrics established for clinical evaluation of conventional venous blood, serum, and plasma samples (e.g., from NHANES, NEJM, etc.).

Lipids: CHO, HDL, TRG

A DBS disc was eluted in a microtiter plate well by shaking at room temperature (RT) for 1hr in 400µL phosphate buffered saline (PBS). To quantify HDL, 40µL DBS eluate was incubated at RT for 2hr with 75µL Reagent 1 (Beckman Coulter). 50µL of Reagent 2 (Beckman Coulter) with 0.01% 10-acetyl-3,7-dihydroxyphenoxazine (ADHP; Cayman Chemical, Ann Arbor, MI) in dH_2_O was then added. After a 2hr RT incubation, ADHP fluorescence from each well was measured spectrophotometrically, and DBS HDL concentration then determined by interpolation of the fluorescence signal on the standard curve. To quantify CHO, 200µL of 0.1% Triton X-100 in dH_2_O was added to the remaining eluates and shaken at RT for 30min to solubilize lipid fractions. 20µL eluate was incubated at RT for 1hr in 100µL Cholesterol reagent (Synermed, Westfield, IN) with 0.01% ADHP, ADHP fluorescence measured, and DBS CHO concentrations then determined by curve interpolation as above. To quantify TRG, 40µL eluate was incubated at RT for 1hr with 100µL Triglyceride reagent (Beckman Coulter) with 0.01% ADHP, ADHP fluorescence measured, and DBS TRG concentrations then determined by curve interpolation as above.

CRP

For the CRP assay, one DBS disc was eluted by shaking at RT for 1hr in 400µL vendor-supplied diluent. CRP was measured in 10µL eluate by ELISA (BioCheck, South San Francisco, CA).

HbA1c

One DBS disc was eluted by shaking at RT for 1hr in 300µL Hemolyzing Solution (Beckman Coulter). HbA1c of the eluate was measured by the DxC 600.

**References**

Brindle E, Fujita M, Shofer J, O'Connor KA. Serum, plasma, and dried blood spot high-sensitivity C-reactive protein enzyme immunoassay for population research. J Immunol Methods. 2010 Oct 31;362(1-2):112-20. doi: 10.1016/j.jim.2010.09.014. Epub 2010 Sep 17. PMID: 20850446; PMCID: PMC2964394

Corso G, Papagni F, Gelzo M, Gallo M, Barone R, Graf M, Scarpato N, Dello Russo A. Development and Validation of an Enzymatic Method for Total Cholesterol Analysis Using Whole Blood Spot. J Clin Lab Anal. 2016 Sep;30(5):517-23. doi: 10.1002/jcla.21890. Epub 2015 Oct 29. PMID: 26511311; PMCID: PMC6807098.

Crimmins E, Kim JK, McCreath H, Faul J, Weir D, Seeman T. Validation of blood-based assays using dried blood spots for use in large population studies. Biodemography Soc Biol. 2014;60(1):38-48. doi: 10.1080/19485565.2014.901885. PMID: 24784986; PMCID: PMC4117354.

Lakshmy R, Mathur P, Gupta R, Shah B, Anand K, Mohan V, Desai NG, Mahanta J, Joshi PP, Thankappan KR. Measurement of cholesterol and triglycerides from a dried blood spot in an Indian Council of Medical Research-World Health Organization multicentric survey on risk factors for noncommunicable diseases in India. J Clin Lipidol. 2012 Jan-Feb;6(1):33-41. doi: 10.1016/j.jacl.2011.10.021. Epub 2011 Nov 12. PMID: 22264572.

Roberts AJ, Malik F, Pihoker C, Dickerson JA. Adapting to telemedicine in the COVID-19 era: Feasibility of dried blood spot testing for hemoglobin A1c. Diabetes Metab Syndr. 2021 Jan-Feb;15(1):433-437. doi: 10.1016/j.dsx.2021.02.010. Epub 2021 Feb 9. PMID: 33588200; PMCID: PMC7871808.

## 2. GM DNA Extraction Protocol for Qiagen PowerSoil PRO DNA Extraction Kit

**Protocol: Detailed**

Important notes before starting:

- Ensure that the PowerBead Pro Tubes rotate freely in the centrifuge without rubbing.
- If Solution CD3 has precipitated, heat at 60°C until precipitate dissolves.
- Perform all centrifugation steps at room temperature (15–25°C).

**Procedure**

1. **Prepare (Needed materials: Get X+1(number of samples) twist tubes with beads in them, 2(X+1) normal flip top, X+1 spin column tubes, X+1 1.5 tubes. CD1,2,3,5,6; EA reagents, 1000 uL and 100 uL filtered tips, 1 small 5 ml tube, X spatulas, 5 big tubes)**
   a. Label all but the 1.5 tubes and the extra small tube 1-X, N for negative
   b. Place in different tube racks Label twist top rack CD1, Normal flip top CD2, Normal Flip top CD3, Filter tube EA/CD5, 1.5 tube CD6.
   c. Label the big tubes CD1, CD2, CD3, EA, CD5. Label the small tube CD6.
   d. Add the proper reagent to the proper tube
   e. Disinfect the spatulas with ethanol and then a Bunsen burner
   f. Lay all tubes on their sides and UV for 30 minutes.
   g. Write which sample corresponds to which numbered tube in the blue notebook. If you have multiple extractions in a day, write which negative is which.
2. **Load tubes with samples (Twist tubes, Solution CD1)**
   a. Spin the PowerBead Pro Tube briefly to ensure that the beads have settled at the bottom.
   b. Add 800 µl of Solution CD1 to each tube, starting with negative.
   c. Add up to 250 mg of fecal matter
   d. Add one empty tube to scale, zero, then weigh each tube after adding fecal matter- should say at least 0.2g
   e. Vortex briefly to mix.
3. **Homogenize samples (CD2 and CD3 aliquots, labeled tubes)**
   a. Leaving the negative, incubate samples at 65 degrees C for 15 minutes. When done, put in CD6 little tube at 60.
   b. Secure the PowerBead Pro Tubes horizontally on a Vortex Adapter for Vortex at maximum speed for 10 min.
   c. Note: If using Vortex Adapter for more than 12 preps simultaneously, increase the vortexing time by 5–10 min.
   d. While incubating/vortexing, prepare CD2 and CD3. Put on new gloves. Starting with the negative, add 200 uL of CD2 to the labeled tubes. Starting with the negative, add 600 of CD3 to labeled tubes.
   e. Centrifuge the PowerBead Pro Tubes at 13, 000 x g for 1 min.
4. **Precipitate Samples (CD2 prepared tubes)**
   a. Avoiding the beads and most of the fecal matter at the bottom, transfer the supernatant to the tubes filled with 200 ul of CD2. Try to get as much as you can

Note: Expect 500–600 µl. The supernatant may still contain some soil particles.

b. Centrifuge at 13,000 x g for 1 min. Incubate in fridge for 10 minutes

5. **Prepare for binding/bind DNA (CD3 prepared tubes, empty spin column tubes, empty 2 mL tubes)**
   a. Avoiding the pellet, transfer up to 700 µl of supernatant to tubes filled with 600 ul of CD3

Note: Expect 500–600 µl.

Note: The pellet at this point contains non-DNA organic and inorganic material including humic acids, cell debris, and proteins. For best DNA yields and quality, avoid transferring any of the pellet.

b. Vortex for 5 s. DO NOT CENTRIFUGE HERE.
c. Avoiding the pellet, transfer up to 700 µl of supernatant to empty MB spin column tubes
d. Centrifuge at 13,000 x g for 30 sec.
e. Discard the flow-through and repeat step 9-10 to ensure that all of the lysate has passed through the MB Spin Column. Get out X+1 empty tubes from kit.
f. Carefully place the MB Spin Column into a clean 2 ml Collection Tube (provided). Avoid splashing any flow-through onto the MB Spin Column.

6. Wash process (Solution EA, C5 aliquot, empty 2 ml tubes)
   a. Add 500 µl of Solution EA to the MB Spin Column. Centrifuge at 13,000 x g for 1 min.
   b. Discard the flow-through and place the MB Spin Column back into the same 2 ml Collection Tube.
   c. Add 500 µl of Solution C5 to the MB Spin Column. Centrifuge at 15,000 x g for 1 min. Get out X+1 empty tubes from kit.
   d. Discard the flow-through and place the MB Spin Column into a new 2 ml Collection Tube (provided).
7. **Dry process (1.5 mL tubes)**
   a. Centrifuge at up to 13,000 x g for 2 min. Label the final 1.5 mL tubes with the actual sample names and the special negative name, if needed.
   b. Carefully place the MB Spin Column into the labeled 1.5 ml Elution Tube (provided).
8. **Release DNA from filter- Elution (Solution CD6)**
   a. Add 100 µl of Solution C6 to the center of the white filter membrane.
   b. Rest on table for 2 min.
   c. Centrifuge at 15,000 x g for 2 min. Cap faces the inside of the centrifuge. Discard the MB Spin Column. The DNA is now ready for downstream applications.
9. **Clean Up**
   a. Place samples in freezer. Discard waste. Wash catch beakers, spatulas with detergent and place in drying rack. Wash inside hood with water, then ethanol. Turn off all equipment.

| *Table S1.* **Health Risk Indicators by Circadian Rhythm Indicators (All Variables Included)** | | | | | | | | | | |
| --- | --- | --- | --- | --- | --- | --- | --- | --- | --- | --- |
|  | **High Sensitivity CRP** | | | **Triglycerides** | | | **Blood Pressure** | | | |
| **Characteristic** | **Beta** | **95% CI**^1^ | **p-value^2^** | **Beta** | **95% CI**^1^ | **p-value^2^** | **Beta** | **95% CI**^1^ | **p-value^2^** |  |
| Age(years) | -0.15 | -0.54, 0.25 | 0.5 | 0.12 | -0.66, 0.89 | 0.8 | -0.13 | -0.34, 0.07 | 0.2 |  |
| Exercise Per Week | -4.3 | -10, 1.8 | 0.2 | -15 | -27, -3.0 | **0.015*** | -3.5 | -6.7, -0.30 | **0.033*** |  |
| Waketime exhaustion from disrupted sleep (incidents per week) | 9.2 | 2.6, 16 | **0.008**** | 0.95 | -12, 14 | 0.9 | -1.3 | -4.7, 2.1 | 0.5 |  |
| Diet Quality (Diet Inflammatory Index) | 0.63 | -2.1, 3.3 | 0.6 | -2.6 | -7.9, 2.8 | 0.3 | 0.35 | -1.0, 1.7 | 0.6 |  |
| Average time between meals | 0.01 | -0.04, 0.05 | 0.7 | 0.00 | -0.09, 0.08 | >0.9 | -0.02 | -0.04, 0.01 | 0.2 |  |
| Time spent on meal breaks (min) | -0.04 | -0.39, 0.31 | 0.8 | 0.09 | -0.60, 0.78 | 0.8 | -0.19 | -0.37, -0.01 | **0.037*** |  |
| Day or Night Shift |  |  |  |  |  |  |  |  |  |  |
| Day | — | — |  | — | — |  | — | — |  |  |
| Night | 9.2 | -2.2, 21 | 0.11 | 3.1 | -19, 26 | 0.8 | 7.0 | 1.2, 13 | **0.019*** |  |
| Sigma | 16.7 |  |  | 32.9 |  |  | 8.39 |  |  |  |
| R² | 0.268 |  |  | 0.150 |  |  | 0.359 |  |  |  |
| No. Obs. | 55 |  |  | 55 |  |  | 52 |  |  |  |
| ^1^CI = Confidence Interval  ^2^*p<0.05; **p<0.01 | | | | | | | | | | |

*Table S2.* **Microbiome PERMANOVA Results Testing CRD Variable Associations with UniFrac Score**

|  | **Df** | **SumOfSqs** | **Rsq (%)** | **F-stat** | **P-value** |
| --- | --- | --- | --- | --- | --- |
| **Unifrac Type: Weighted** | | | | | |
| Age(years) | 1 | 0.03853295 | 1.452532 | 0.9875406 | 0.47 |
| Exercise Incidents Per Week | 3 | 0.16194034 | 6.104477 | 1.4086258 | 0.096 |
| Day vs Night Shift | 1 | 0.03653026 | 1.377039 | 0.9354982 | 0.483 |
| Workplace exhaustion level from lack of sleep | 1 | 0.03257807 | 1.256959 | 0.8401533 | 0.603 |
| Diet Quality (Diet Inflammatory Index) | 3 | 0.10545795 | 4.404199 | 0.9214211 | 0.597 |
| Average time between meals | 1 | 0.03903959 | 1.780300 | 0.9787873 | 0.434 |
| Time spent eating meals at work (min) | 1 | 0.04675691 | 1.762541 | 1.2020898 | 0.248 |
| **Unifrac Type: Unweighted** | | | | | |
| Age(years) | 1 | 0.23041863 | 1.435355 | 0.9756928 | 0.529 |
| Exercise Incidents Per Week | 3 | 0.78629159 | 4.898076 | 1.1159077 | 0.082 |
| Day vs Night Shift | 1 | 0.27178324 | 1.693029 | 1.1538650 | 0.122 |
| Workplace exhaustion level from lack of sleep | 1 | 0.23525955 | 1.490387 | 0.9985376 | 0.456 |
| Diet Quality (Diet Inflammatory Index) | 3 | 0.72948493 | 4.928392 | 1.0367748 | 0.320 |
| Average time between meals | 1 | 0.22376613 | 1.718843 | 0.9444079 | 0.665 |
| Time spent eating meals at work (min) | 1 | 0.21844573 | 1.360772 | 0.9242949 | 0.692 |
